# Glucose 6-phosphate dehydrogenase knockdown enhances IL-8 expression in HepG2 cells via oxidative stress and NF-κB signaling pathway

**DOI:** 10.1186/s12950-015-0078-z

**Published:** 2015-04-24

**Authors:** Hung-Chi Yang, Mei-Ling Cheng, Yi-Syuan Hua, Yi-Hsuan Wu, Hsin-Ru Lin, Hui-Ya Liu, Hung-Yao Ho, Daniel Tsun-Yee Chiu

**Affiliations:** Healthy Aging Research Center, Chang Gung University, Kwei-Shan, Tao-Yuan 333 Taiwan; Department of Medical Biotechnology and Laboratory Sciences, College of Medicine, Chang Gung University, Kwei-Shan, Tao-Yuan 333 Taiwan; Department of Biomedical Sciences, College of Medicine, Chang Gung University, Kwei-Shan, Tao-Yuan 333 Taiwan; Department of Clinical Pathology, Chang Gung Memorial Hospital, Kwei-Shan, Tao-Yuan 333 Taiwan; Molecular Medicine Research Center, College of Medicine, Chang Gung University, Kwei-Shan, Tao-Yuan 333 Taiwan

**Keywords:** G6PD deficiency, Oxidative stress, Pro-inflammatory cytokine, IL-8, Palmitate, Antioxidant, NF-κB

## Abstract

**Background:**

This study was designed to investigate the effect of glucose 6-phosphate dehydrogenase (G6PD) deficiency on pro-inflammatory cytokine secretion using a palmitate-induced inflammation HepG2 in vitro model. The modulation of cellular pro-inflammatory cytokine expression under G6PD deficiency during chronic hepatic inflammation has never been investigated before.

**Methods:**

The culture medium of untreated and palmitate-treated G6PD-scramble (Sc) and G6PD-knockdown (Gi) HepG2 cells were subjected to cytokine array analysis, followed by validation with ELISA and qRT-PCR of the target cytokine. The mechanism of altered cytokine secretion in palmitate-treated Sc and Gi HepG2 cells was examined in the presence of anti-oxidative enzyme (glutathione peroxidase, GPX), anti-inflammatory agent (curcumin), NF-κB inhibitor (BAY11-7085) and specific SiRNA against NF-κB subunit p65.

**Results:**

Cytokine array analysis indicated that IL-8 is most significantly increased in G6PD-knockdown HepG2 cells. The up-regulation of IL-8 caused by G6PD deficiency in HepG2 cells was confirmed in other G6PD-deficient cells by qRT-PCR. The partial reduction of G6PD deficiency-derived IL-8 due to GPX and NF-κB blockers indicated that G6PD deficiency up-regulates pro-inflammatory cytokine IL-8 through oxidative stress and NF-κB pathway.

**Conclusions:**

G6PD deficiency predisposes cells to enhanced production of pro-inflammatory cytokine IL-8. Mechanistically, G6PD deficiency up-regulates IL-8 through oxidative stress and NF-κB pathway. The palmitate-induced inflammation in G6PD-deficient HepG2 cells could serve as an in vitro model to study the role of altered redox homeostasis in chronic hepatic inflammation.

**Electronic supplementary material:**

The online version of this article (doi:10.1186/s12950-015-0078-z) contains supplementary material, which is available to authorized users.

## Background

Glucose 6-phosphate dehydrogenase (G6PD) catalyzes the rate-limiting step in the hexose monophosphate shunt with the concomitant generation of reduced nicotinamide adenine dinucleotide phosphate (NADPH), which is involved in cellular reductive biosynthesis and redox homeostasis [1]. G6PD deficiency, also known as favism, is one of the most common genetic disorders in the world affecting an estimated four hundred million people worldwide [2]. G6PD is the only enzyme in erythrocytes to regenerate NADPH and subsequently glutathione (GSH), which protects red cells against oxidative attacks. G6PD-deficient erythrocytes are particularly susceptible to hemolysis upon exposure to oxidants such as fava beans or primaquine. Classically, since G6PD deficiency has been associated with hemolytic crise as a major clinical manifestation [3], most studies on G6PD deficiency have been focused on erythrocytes [4]. Recently, an increasing number of studies have shown that G6PD deficiency not only affects erythrocytes, but also induces aberrations of cellular functions in nucleated cells [5-11].

Redox status plays an essential role in the modulation of inflammation and immune response [12]. During inflammation, the production of intracellular reactive oxygen species (ROS) is involved in triggering inflammatory responses via the secretion of pro-inflammatory cytokines, which can directly affect the course of inflammation-associated diseases [13,14]. Among the pro-inflammatory cytokines, Interleukin-8 (IL-8) has received much attention owing to its capacity to mediate polymorphonuclear neutrophils (PMN) chemotaxis through CXC chemokine receptor 1 (CXCR1) and 2 (CXCR2) [15]. Several stimulating factors are known to up-regulate the expression of IL-8, including lipopolysaccharide (LPS) [16], phytohemagglutinin (PHA) [17], aggregated immune complex (IC) [18], tumor necrosis factor (TNF) [19], Interleukin-1β (IL-1 β) [20] and palmitate [21-24]. The activation of nuclear factor kappa B (NF-κB), NF-IL-6 (C/EBP β) or activator protein-1 (AP-1) is required for the transcription of IL-8 [25-27]. Although redox status is known to modulate cytokines, the relationship between G6PD and pro-inflammatory cytokines, such as IL-8, has been overlooked. Further investigation is warranted for how G6PD modulates pro-inflammatory cytokines and affects the inflammatory response.

Inflammation is a critical component in different types of acute and chronic liver disorders, which consequently progress to hepatitis and fibrosis [28]. In this study, we have adopted a palmitate-induced inflammation HepG2 in vitro model [29] and performed cytokine array analysis to investigate the role of G6PD during chronic hepatic inflammation. We have shown that G6PD modulates the secretion of the pro-inflammatory cytokine IL-8 via oxidative stress and NF-κB pathway because the elevated IL-8 due to G6PD deficiency is partially blocked by exogenous antioxidant and NF-κB inhibitors. These findings suggest that G6PD may play an important role in the modulation of chronic hepatic inflammation.

## Methods

### Materials

Dulbecco’s modified Eagle’s medium (DMEM), fetal calf serum (FCS), streptomycin, and penicillin were purchased from Invitrogen (CA, USA). Hydrogen peroxide was obtained from Merck (Darmstadt, Germany). BAY11-7085 (NF-κB inhibitor) was purchased from Calbiochem (Millipore, MA, USA). Glutathione peroxidase, curcumin and human SiRNA of scrambled control and p65, were purchased from Sigma (MO, USA)

### Cell culture

Human hepatocarcinoma cell lines HepG2, SK-Hep1 and the primary human foreskin fibroblast (HFF) were grown in DMEM supplemented with 10% FCS, antibiotics (100 units/ml penicillin and 100 mg/ml streptomycin) and 5% CO_2_ at 37°C. The construction and generation of the G6PD scramble control (Sc, G6PD normal) and G6PD shRNA (Gi, G6PD-deficient) HepG2 cells [7,30] as well as G6PD scramble control (SK-i-Sc, G6PD normal) and G6PD shRNA (SK-i-Gi, G6PD-deficient) SK-Hep1 cells [11] were described previously. The isolation and the characterization of normal primary HFF (HFF3, G6PD normal) and its G6PD-deficient HFF counterpart (HFF1, G6PD-deficient) were reported previously [31].

### Induction of palmitate overload in HepG2 cells

The induction of palmitate overload in HepG2 cells was performed according to a published protocol [29]. In brief, 3×10^6^ HepG2 cells were cultured in petridish for overnight at 37°C. The culture medium was then replaced with fresh medium containing palmitate and the cells were continued to culture for 24 hr. The palmitate-containing medium was prepared by diluting 150 mM palmitate stock solution (dissolved in isopropanol) to DMEM supplemented with 1% fatty acid free bovine serum albumin (Sigma) followed by overnight incubation at 37°C.

### Cell viability assay

The HepG2 cell viability was measured based on the Neutral Red uptake assay described previously [6]. In brief, 1.2×10^5^ untreated or palmitate-treated Sc and Gi HepG2 cells were cultured in 24-well plates for overnight at 37°C. At the end of treatment, cells were incubated with 0.5 ml of 0.033% Neutral Red solution for 2 hr at 37°C followed by fixation with 0.5 ml of 0.1% CaCl_2_ and 0.5% formaldehyde for 5 min. Subsequently, the incorporated dye was solubilized in 0.5 ml of 1% acetic acid and 50% ethanol solution. The absorbance of Neutral Red was detected at 540 nm with a reference wavelength at 690 nm using a SpectraMax 340PC384 microplate reader (Molecular Devices, CA, USA).

### ROS measurement and ROS treatment

The ROS level of palmitate-treated Sc and Gi HepG2 cells was analyzed by flow cytometry based on a previous protocol [6]. In brief, 3×10^6^ HepG2 cells were cultured in petridish for overnight at 37°C. After palmitate treatment, cells were incubated with 20 μM 2′, 7′-dichlorodihydrofluoroscein diacetate (DCF-DA) solution (Molecular Probes, OR, USA) for 30 min at 37°C followed by Trypsin-EDTA treatment. The trypsinized cells were then detected for ROS production by a FACS Calibur flow cytometer (Becton Dickson, CA, USA) (excitation 490 nm, emission 520 nm). The data was analyzed by Cell Quest Pro software (Becton Dickson). For ROS treatment, 3×10^6^ Sc and Gi HepG2 cells were seeded separately in petridish. After 24 hr, the medium was replaced by fresh medium with or without 0.5 mM H_2_O_2_ and continued to culture for 24 hr. After the treatment, the medium of Sc and Gi HepG2 cells were collected for the determination of IL-8 secretion.

### Gene expression by quantitative real-time PCR

The quantitative real-time PCR (qRT-PCR) was conducted by using SYBR Green PCR Premix reagent (Yeastern Biotechnology, Taipei, Taiwan) with iQ5 real-time thermal cycler (Bio-Rad, CA, USA) according to a previous publication [32]. Primers were designed using Beacon designer software (Bio-Rad). In brief, HepG2 (3×10^6^ cells), SK-Hep1 (3×10^6^ cells), and primary human foreskin fibroblasts (5×10^5^ cells) were seeded separately in petridish and cultured for 3 days until harvesting for RNA isolation. The RNA was extracted by using TRIzol reagent (Life Technologies, CA, USA) according to the instructions provided by the manufacturer. The concentration of RNA was quantified by Nanophotometer (Implen, Munich, Germany). 1 μg of RNA was reversely transcribed to cDNA by using SuperScript III Reverse Transcriptase (Life Technologies) with 0.5 μg of oligo (dT)_18_ primer (Bioman Scientific, Taipei, Taiwan). The qRT-PCR reaction contained 1 μg of cDNA, primers, and SYBR Green PCR Premix. The thermal cycle program was set as followed: 95°C for 10 min, 40 cycles of 95°C for 15 sec and 60°C for 1 min. The gene expression of IL-8 (forward primer: 5′-CTTTCAGAGACAGCAGAG-3′; reverse primer: 5′-CTAAGTTCTTTAGCACTCC-3′) was normalized against threshold cycle (Ct) values of the housekeeping gene actin (forward primer: 5′-TCCACCTTCCAGCAGATG-3′; reverse primer: 5′-GTGTAACGCAACTAAGTCATAG-3′). The relative index (2^**-**ΔΔCt^) was calculated by comparing the average expression level for control samples with the index defined as 1.00.

### Cytokine profiling

The cytokine profiling of Sc and Gi HepG2 cells with or without palmitate treatment was determined by using Human Cytokine Array Panel A, Proteome Profiler™ Array (R&D systems). In brief, 3×10^6^ HepG2 cells were cultured in petridish for overnight followed by treating with or without palmitate for 24 hr at 37°C. The culture medium of each sample was collected and concentrated with 5 kDa centrifugal filter units (Millipore, MA, USA). The volume of concentrated medium was adjusted to 1 ml followed by mixing with Cytokine Array Detection Antibody Cocktail for 1 hr at room temperature. The concentrated medium/antibody mixture was then incubated with the nitrocellulose membrane, which contains 36 different anti-cytokine antibodies printed in duplicate, for overnight at 4°C on a rocking shaker (Firstek Scientific, Taiwan). The washed membrane was incubated with diluted Streptavidin-Horseradish Peroxidase (HRP) for 30 min at room temperature on a rocking shaker. The membrane was washed and incubated with chemiluminescent detection reagent for 1 min. Lastly, the membrane was covered with plastic wrap in a X-ray film cassette and exposed to X-ray film (Fujifilm, Japan). The cytokine array data on developed a X-ray film was quantified by scanning the film on a transmission-mode scanner. The array image was analyzed by image analysis software (Image J). The average signal (pixel density) of duplicate spots representing each cytokine was obtained by subtracting averaged background signal on the array. In order to obtain the relative level of each cytokine between different samples, the IL-8 protein level of each sample was first quantified by ELISA. The level of IL-8 was then defined as a reference to which other cytokines were normalized in each array image. After normalization to IL-8, comparison of the signal intensity of each cytokine between array images can be used to determine the relative level of each cytokine in different samples.

### Determination of IL-8 by ELISA

The IL-8 protein level in the culture medium secreted by Sc and Gi HepG2 cells with or without palmitate treatment was quantified by the human IL-8 enzyme-linked immunosorbent assay (ELISA) kit (R&D systems). In brief, 3×10^6^ HepG2 cells were cultured in petridish for overnight followed by treating with or without palmitate for 24 hr at 37°C. The culture medium of each sample was collected and concentrated with 5 kDa centrifugal filter units (Millipore). The concentrated medium of each sample and IL-8 standard (50 μl) were transferred to the wells containing 100 μl assay diluent in a flat-bottomed 96-well plate coated with IL-8 antibody and incubated for 2 hr at room temperature. The plate was aspirated and washed followed by adding 100 μl IL-8 conjugate (IL-8 antibody conjugated to HRP) for 1 hr at room temperature. The plate was then aspirated and washed for four times. The working solution of Streptavidin-HRP was added subsequently and incubated for 30 min at room temperature followed by aspiration. Lastly, the stabilized chromogen was added to the plate for 30 min at room temperature in the dark followed by adding stop solution. The absorbance of each well was detected at 450 nm with a reference at 490 nm in a microplate reader (Versa MAX, Molecular Devices). The recombinant human IL-8 protein (R&D systems) was used for the generation of a standard curve for IL-8.

### Small interfering RNA (SiRNA)

3×10^5^ HepG2 cells were cultured in a 6-well plate for overnight at 37°C. The culture medium was subsequently replaced with fresh DMEM and incubated for 1 hr before SiRNA transfection. The cells were transfected with SiRNA oligonucleotides using 7.5 μl PolyJet In Vitro DNA Transfection Reagent (SignaGen Laboratories, MD, USA). Two different SiRNA oligonucleotides were used: (1) SiRNA against non-targeting scramble control, (2) SiRNA against human NF-κB subunit p65. Both Sc and Gi HepG2 cells were transfected with SiRNA-control and SiRNA-p65 in different concentrations (50, 100, 150 nM) in a total volume of 1 ml DMEM per well. After 72 hr incubation, the culture medium of each well was collected for the determination of IL-8 secretion by ELISA. The cells in each well were harvested for isolation of RNA and protein for measuring IL-8 mRNA and p65 level by qRT-PCR and Western blotting, respectively.

### Western blot analysis

The protein lysate isolated from cells was resolved by SDS-PAGE (TGX Fastcast, Bio-Rad) and electrotransferred by semi-dry Western blotting (Trans-Blot Turbo blotting system, Bio-Rad). The immunoblotting was conducted by using antibody against human p65 (Cell Signaling technology, MA, USA), actin (Santa Cruz Biotechnology, TX, USA) according to the protocols provided by manufacturers.

### Statistical analysis

Data were presented as means ± S.D. Student’s t-test was used to analyze the statistical difference between G6PD-scramble (G6PD normal) and G6PD-knockdown (G6PD-deficient) HepG2 cells. Comparisons between different concentrations or time course of palmitate treatment were evaluated by one-way analysis of variance followed by Tukey’s multiple comparison test. Value of *P* < 0.05 was considered statistically significant.

## Results

### Establishment of a G6PD-knockdown HepG2 cell model with or without palmitate treatment

To investigate the role of G6PD in chronic hepatic inflammation, we adopted a stable G6PD-knockdown HepG2 cell model [6] undergoing lipid-induced inflammation [29]. Consistent with previous report, the G6PD-knockdown (Gi) HepG2 cells displayed a significant reduction (10-fold, *P* < 0.05) of G6PD catalytic activity (see Additional file 1 for all supplementary materials; Additional file 2: Figure S1a) as well as protein expression (Additional file 2: Figure S1b) compared to its G6PD normal counterpart, G6PD-scramble (Sc) HepG2 cells. Upon palmitate treatment, both Sc and Gi HepG2 cells showed an increase of lipid deposition as indicated by microscopic examination with Sudan Red staining (Additional file 3: Figure S2). This observation was corroborated by flow cytometry analysis with Nile Red staining (Additional file 4: Figure S3). To address whether palmitate treatment induced cytotoxicity in HepG2 cell upon G6PD deficiency, the cell viability was determined in control and palmitate-treated Sc and Gi HepG2 cells by Neutral Red uptake assay (Table 1). The result showed that the viability of Sc and Gi HepG2 cells was reduced by palmitate treatment in a dose-dependent fashion. At 0.4 and 0.6 mM palmitate, there was a significant difference of viability between Sc and Gi HepG2 cells (*P* < 0.05). These data demonstrate that in the palmitate-treated cell model, G6PD deficiency does not affect lipid accumulation, but renders cells more susceptible to cytotoxic effect at higher palmitate concentrations.

**Table 1:** **The effect of palmitate treatments on the viability of HepG2 cells**

**[Palmitate] mM**	**0**	**0.2**	**0.4**	**0.6**
Sc	100%	90%	60%	35%
Gi	100%	91%	50%*	25%*

The viability of palmitate-treated cells was reported in percentage. The data represent the mean of at least three separate experiments. * indicates a significant difference (*P* < 0.05) between Sc and Gi HepG2 cells.

### Enhancement of pro-inflammatory cytokine secretion in G6PD-knockdown HepG2 cells

**Figure 1:**
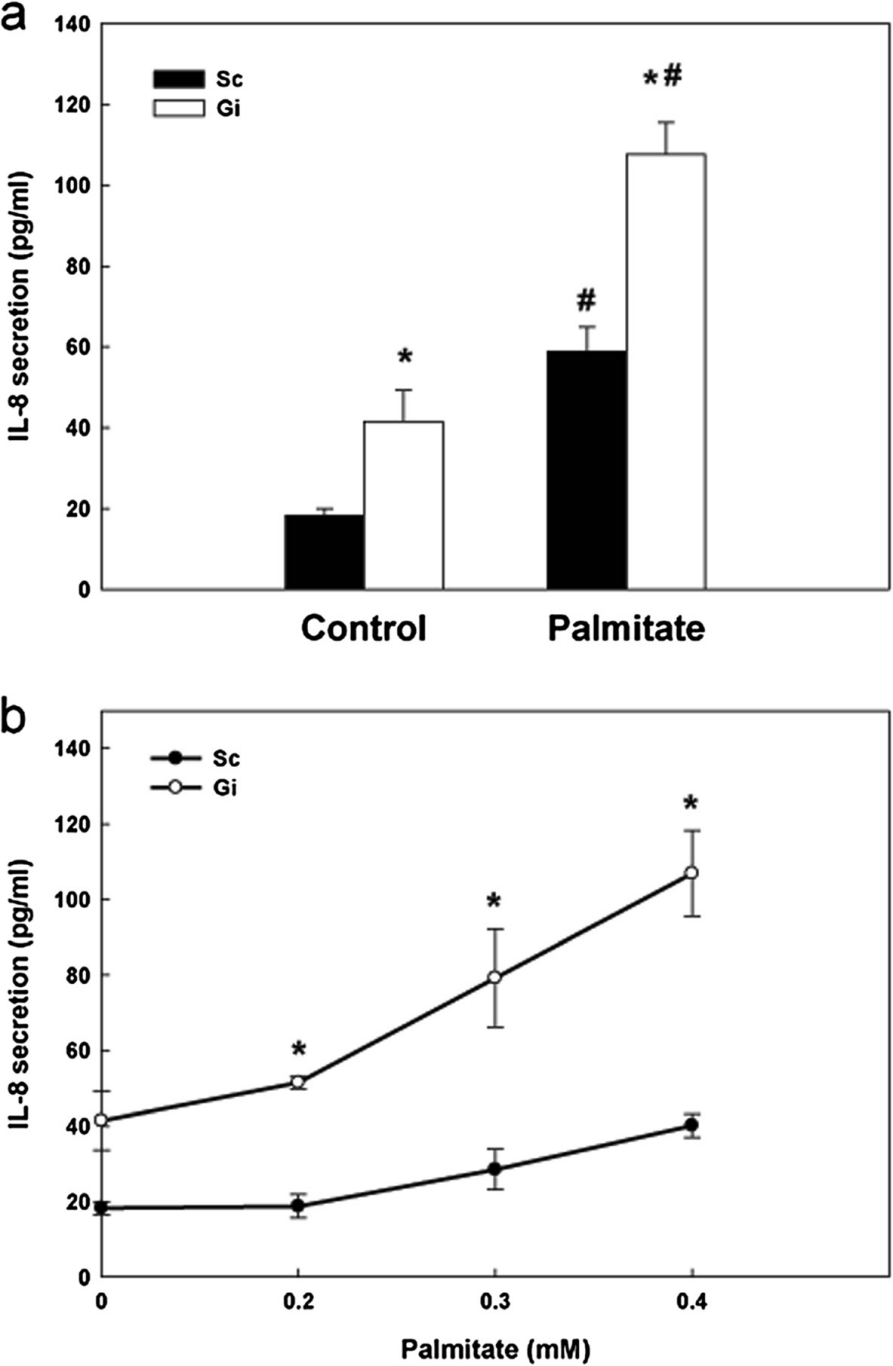
The effect of G6PD deficiency on IL-8 secretion in palmitate-treated HepG2 cells. **(a)** The IL-8 secretion of Sc and Gi HepG2 cells with or without palmitate treatment (0.6 mM, 24 hr) was measured by ELISA. **(b)** The IL-8 secretion of Sc and Gi HepG2 cells treated with different concentrations of palmitate (0.2, 0.3, 0.4 mM) for 24 hr was analyzed by ELISA. These results are representative of at least three separate experiments. *indicates a significant difference (*P* < 0.05) between Sc and Gi HepG2 cells. ^#^indicates a significant difference (*P* < 0.05) between control and palmitate-treated HepG2 cells.

**Figure 2:**
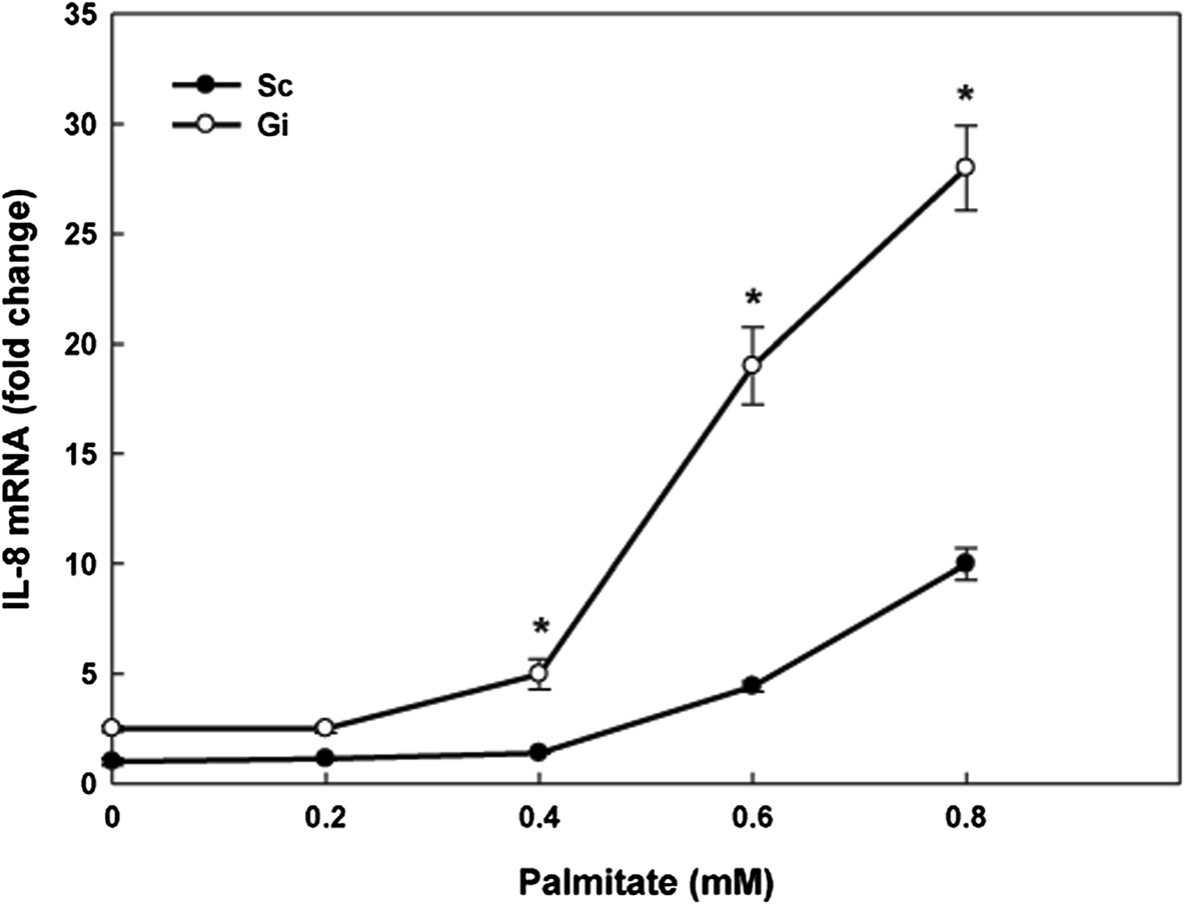
The effect of G6PD deficiency on IL-8 mRNA level in palmitate-treated HepG2 cells. The mRNA level of IL-8 in Sc and Gi HepG2 cells treated with different concentrations of palmitate (0.2, 0.4, 0.6, 0.8 mM) for 24 hr was analyzed by qRT-PCR. These results are representative of at least three separate experiments. *indicates a significant difference (*P* < 0.05) between Sc and Gi HepG2 cells.

Since palmitate induces pro-inflammatory cytokine IL-8 secretion in hepatocytes [21], we investigated whether G6PD deficiency affected pro-inflammatory cytokine secretion in the palmitate-treated cell model by cytokine array analysis. Among 36 different cytokines tested, several cytokines (IL-8, IL-1ra, MIF, sICAM-1, and Serpin E1) were detected (Additional file 5: Figure S4 and see the array format in Additional file 6: Table S1). Interestingly, G6PD deficiency enhanced secretion of these cytokines at basal and palmitate treatment conditions (Table 2). In particular, the IL-8 secretion showed a 9-fold increase in palmitate-treated Gi HepG2 cells compared with untreated Sc HepG2 cells, whereas the secretion of other high level cytokines showed a 5-fold increase. Since IL-8 showed a most dramatic increase in cytokine array, we validated its up-regulation in Gi HepG2 cells by ELISA (Figure 1a) and qRT-PCR (Figure 2). The ELISA data confirmed that IL-8 secretion was significantly increased (*P* < 0.05) in palmitate-treated Gi HepG2 cells (Figure 1a) in a palmitate-dependent manner (Figure 1b). Likewise, the qRT-PCR result also validated that G6PD deficiency significantly up-regulated IL-8 gene expression upon palmitate treatment in a palmitate-dependent manner (Figure 2).

**Table 2:** **List of differentially expressed cytokines of Sc and Gi HepG2 cells in basal and palmitate-treated conditions identified by cytokine array**

**Cytokines**	**Full name**	**Relative level**			
		**Basal**		**Palmitate**	
		**Sc**	**Gi**	**Sc**	**Gi**
IL-8	Interleukin-8	1.00	2.12	3.00	9.18
IL-1ra	Interleukin-1 receptor antagonist	1.00	2.57	2.39	5.16
MIF	Macrophage migration inhibitory factor	1.00	2.37	3.04	5.88
sICAM-1	Soluble intercellular adhesion molecule-1	1.00	2.22	2.64	5.15
Serpin E1	Serpin peptidase inhibitor, clad E	1.00	2.54	2.60	4.92

In palmitate treatment condition, cells were treated with 0.3 mM palmitate for 24 hr.

The relative intensity of each cytokine was normalized to untreated Sc HepG2 cells as 1.00.

### Enhancement of IL-8 expression in other G6PD-deficient cells

**Figure 3:**
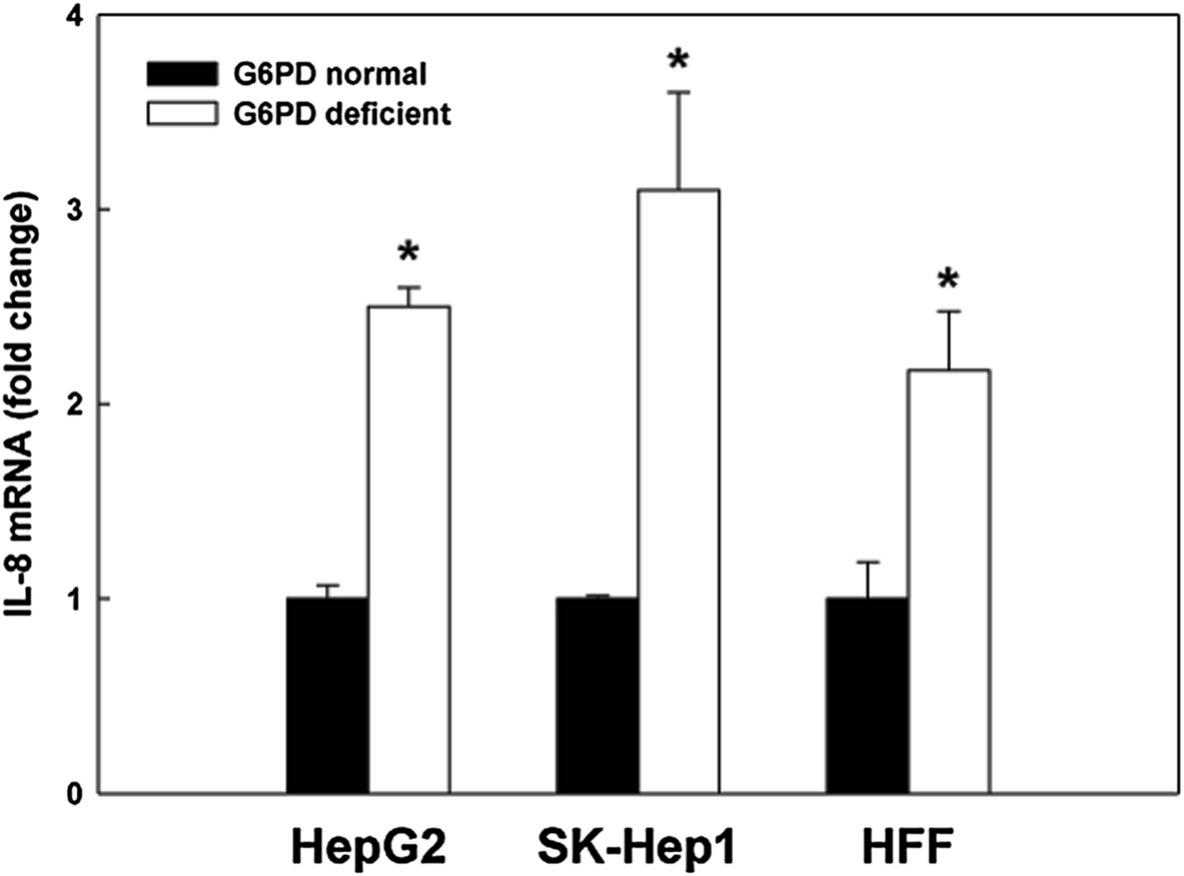
The effect of G6PD deficiency on IL-8 mRNA level in cells. The mRNA level of IL-8 in G6PD normal and G6PD-deficient cells (HepG2, SK-Hep1, and HFF) was determined by qRT-PCR. These results are representative of at least three separate experiments. *indicates a significant difference (*P* < 0.05) between G6PD normal and G6PD-deficient cells.

To rule out the up-regulation of IL-8 induced by G6PD deficiency in HepG2 cell was merely a cell-specific observation, the IL-8 level of other G6PD-deficient cells (SK-Hep1 hepatoma cells and HFF fibroblasts) was analyzed by qRT-PCR (Figure 3). Significantly elevated IL-8 gene expression in G6PD-deficient SK-Hep1 and HFF cells was also observed. These findings suggest that the up-regulation of IL-8 induced by G6PD deficiency is not a cell-specific, but a general phenomenon.

### Involvement of NF-κB in G6PD deficiency induced IL-8 secretion in HepG2 cells

**Figure 4:**
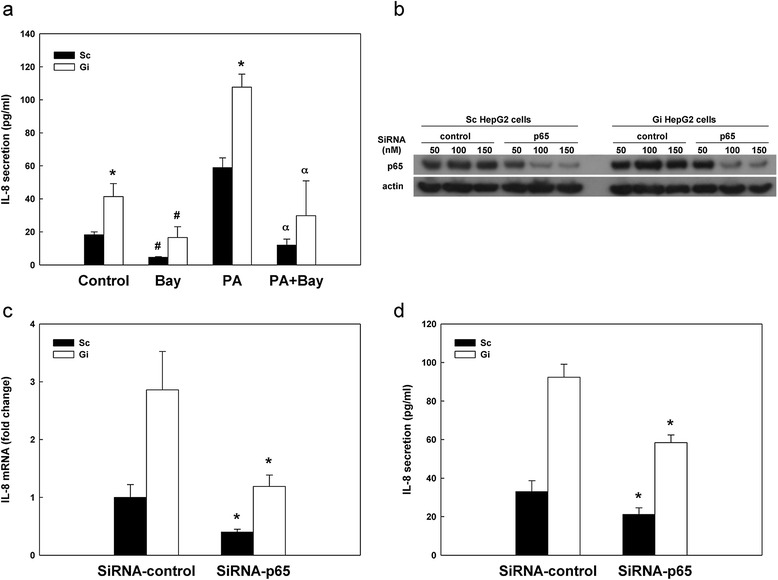
The effect of NF-κB inhibition on IL-8 secretion in HepG2 cells. **(a)** The effect of NF-κB inhibitor on IL-8 secretion with or without palmitate treatment. The IL-8 secretion of Sc and Gi HepG2 cells treated with 10 μM NF-κB inhibitor (BAY11-7085) with or without 0.6 mM palmitate for 24 hr was measured by ELISA. These results are representative of at least three separate experiments. *indicates a significant difference (*P* < 0.05) between Sc and Gi HepG2 cells. ^#^indicates a significant difference (*P* < 0.05) between control and inhibitor (BAY)-treated HepG2 cells without palmitate. α indicates a significant difference (*P* < 0.05) between palmitate (PA)-treated cells without inhibitor (BAY) and palmitate (PA)-inhibitor (BAY) co-treated HepG2 cells. **(b)** The effect of p65 knockdown in Sc and Gi HepG2 cells on p65 protein level determined by Western blotting. The blot (cell lysate: 25 μg protein content) was incubated with anti-p65 antibody and subsequently stripped for incubation with anti-actin antibody as loading control. **(c)** The effect of p65 knockdown on IL-8 gene expression by qRT-PCR and **(d)** IL-8 secretion by ELISA. These results are representative of at least three separate experiments. *indicates a significant difference (*P* < 0.05) between SiRNA- control and SiRNA- p65.

It has been reported that G6PD activates NF-κB in β-cells and adipocytes [33,34]. To investigate whether NF-κB pathway was involved in the enhancement of IL-8 secretion in G6PD-knockdown cells, Sc and Gi HepG2 cells with or without 0.6 mM palmitate treatment were exposed to 10 μM NF-κB inhibitor (BAY11-7085) followed by quantification of IL-8 secretion. As shown in Figure 4a, IL-8 secretion was significantly reduced by the NF-κB inhibitor in Sc HepG2 cells without palmitate treatment (75% decrease, *P* < 0.05) compared to that in untreated control cells. Similarly, the inhibitor reduced the IL-8 secretion in Gi HepG2 cells compared to that in untreated Gi HepG2 cells without palmitate treatment (64% decrease, *P* < 0.05). In the palmitate-treated cells, the NF-κB inhibitor significantly reduced IL-8 secretion (80% decrease, *P* < 0.05) in Sc HepG2 cells. Likewise, the NF-κB inhibitor significantly reduced the palmitate-induced IL-8 secretion in Gi HepG2 cells (72% decrease, *P* < 0.05) compared with palmitate-treated only Gi HepG2 cells. To further investigate the association of G6PD and NF-κB signaling on IL-8 secretion, a G6PD/ NF-κB(p65) double-knockdown cell line was established (Figure 4b). Because the immunoblotting result showed that the protein level of p65 was diminished markedly by SiRNA against p65 at 150 nM, such condition was used for the measurement of IL-8 level. The knockdown of NF-κB(p65) in Sc and Gi HepG2 cells resulted in a significant reduction of IL-8 mRNA (60% decrease, *P* < 0.05) compared with Sc and Gi HepG2 cells treated with control SiRNA (Figure 4c). In addition, Sc and Gi HepG2 cells treated with p65 SiRNA exhibited diminished IL-8 secretion (40% decrease, *P* < 0.05) compared to Sc and Gi HepG2 cells treated with control SiRNA (Figure 4d). The blockade of NF-κB due to selective inhibitor or specific SiRNA against NF-κB confirms that the up-regulation of IL-8 caused by G6PD deficiency is linked to NF-κB signaling. Nevertheless, the finding that the inhibition of NF-κB can not fully suppress the elevated IL-8 in Gi HepG2 cells suggests that besides NF-κB pathway there maybe alternative regulatory mechanism modulated by G6PD.

### Enhancement of IL-8 production by H_2_O_2_

**Figure 5:**
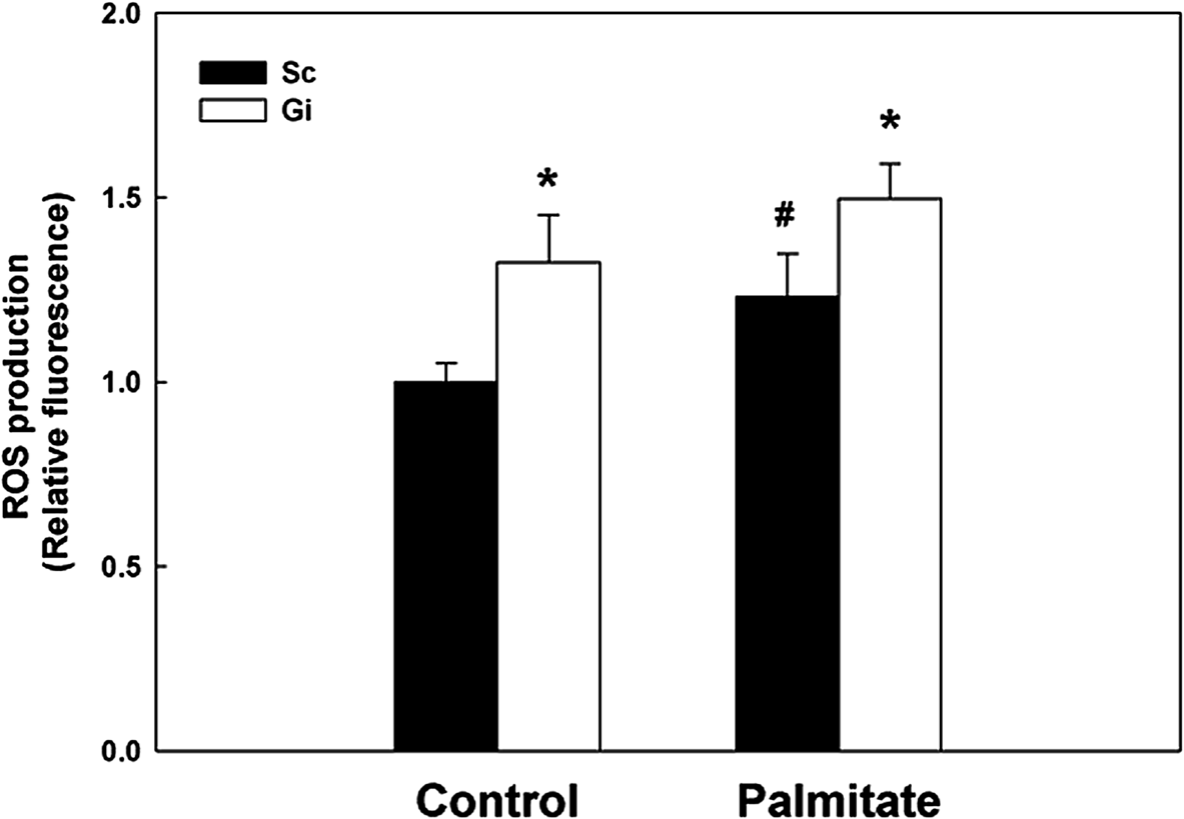
The effect of G6PD deficiency on ROS production in HepG2 cells with or without palmitate treatment. The ROS production of palmitate-treated (0.3 mM, 24 hr) Sc and Gi HepG2 cells was determined by DCF-DA staining and analyzed by flow cytometry. These results are representative of at least three separate experiments. *indicates a significant difference (*P* < 0.05) between Sc and Gi HepG2 cells. ^#^indicates a significant difference (*P* < 0.05) between control and palmitate-treated HepG2 cells.

**Figure 6:**
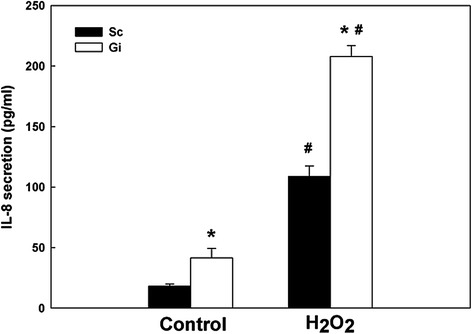
The effect of exogenous hydrogen peroxide on IL-8 secretion in HepG2 cells. The IL-8 secretion of Sc and Gi HepG2 cells with or without treatment of 0.5 mM H_2_O_2_ for 24 hr was measured by ELISA. These results are representative of at least three separate experiments. *indicates a significant difference (*P* < 0.05) between Sc and Gi HepG2 cells. ^#^indicates a significant difference (*P* < 0.05) between control and H_2_O_2_-treated HepG2 cells.

G6PD-deficient HepG2 cells are extremely sensitive to cytotoxic effects induced by oxidants with the concomitant production of oxidative stress [6,7]. To determine whether the enhanced IL-8 expression in palmitate-treated Gi HepG2 cells was due to oxidative stress, the ROS level of Sc and Gi HepG2 cells in control and palmitate-treated conditions was determined. As shown in Figure 5, Gi HepG2 cells without palmitate treatment displayed a 1.4-fold increase of ROS level (*P* < 0.05) compared with that of control Sc HepG2 cells, while in palmitate-treated Sc and Gi HepG2 cells there was an increase in ROS over that in control with a similar ratio between the two cell types. To examine the effect of oxidative stress on IL-8 secretion, both Sc and Gi HepG2 cells were treated with 0.5 mM H_2_O_2_ added exogenously according to a previous protocol [6] followed by quantification of IL-8 secretion. As shown in Figure 6, H_2_O_2_ treatment significantly enhanced IL-8 secretion in both Sc and Gi HepG2 cells (5-fold increase, *P* < 0.05) compared with that in control cells. The dramatic increased in IL-8 secretion by H_2_O_2_, particularly in Gi HepG2 cells, is consistent with the notion that IL-8 secretion in HepG2 cells is highly sensitive to oxidative stress.

### Modulation of IL-8 production by glutathione peroxidase and curcumin

**Figure 7:**
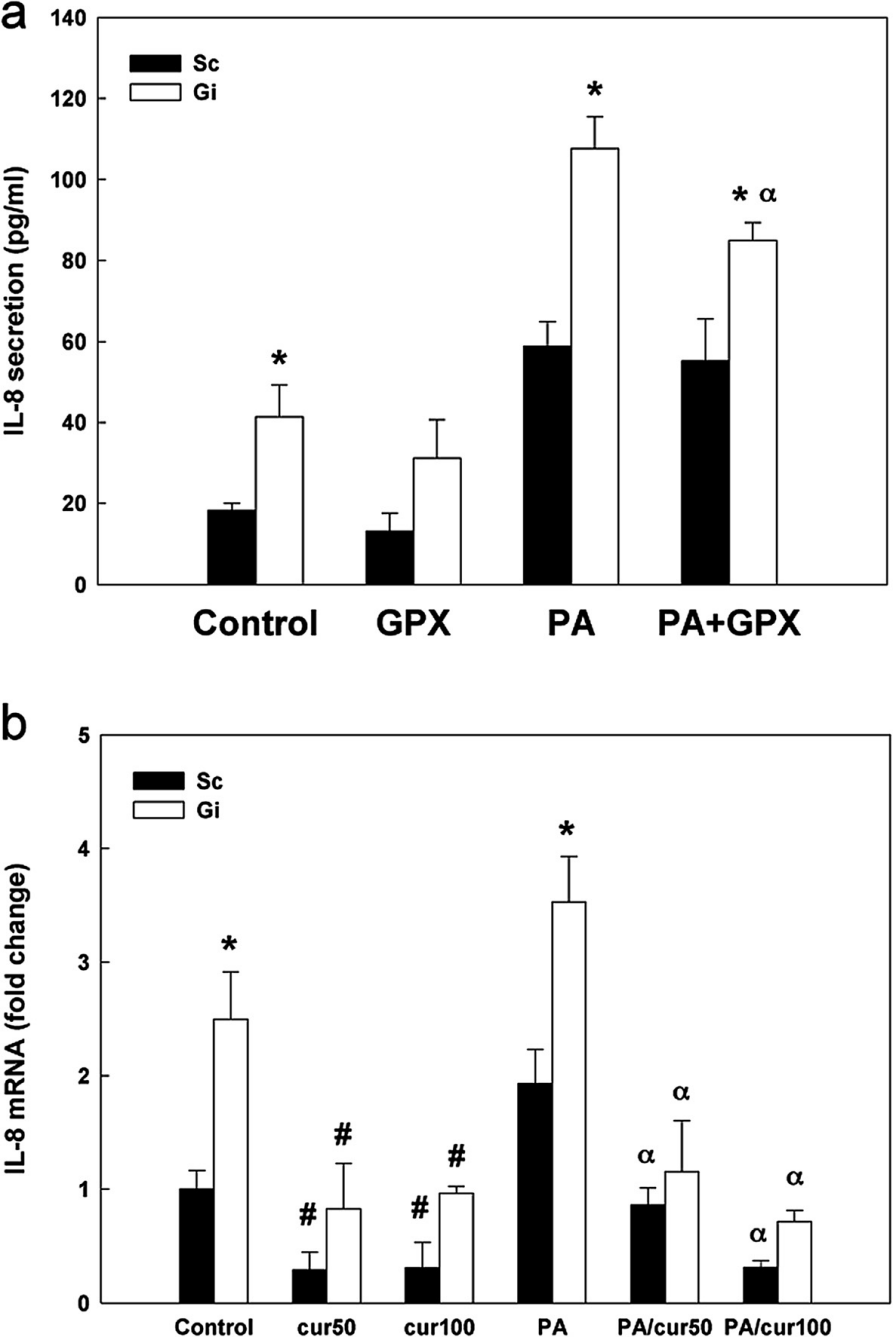
The effect of GPX and curcumin on IL-8 secretion in Sc and Gi HepG2 cells with or without palmitate treatment. **(a)** The IL-8 secretion of Sc and Gi HepG2 cells co-treated with 2 U/ml glutathione peroxidase (GPX) and 0.6 mM palmitate (PA) for 24 hr was measured by ELISA. These results are representative of at least three separate experiments. *indicates a significant difference (*P* < 0.05) between Sc and Gi HepG2 cells. α indicates a significant difference (*P* < 0.05) between PA without GPX and PA-GPX co-treated HepG2 cells. **(b)** The IL-8 mRNA of Sc and Gi HepG2 cells co-treated with 50 or 100 μΜ curcumin (referred to cur50 and cur100, respectively) and 0.6 mM palmitate (PA) for 6 hr was measured by qRT-PCR. These results are representative of at least three separate experiments. *indicates a significant difference (*P* < 0.05) between Sc and Gi HepG2 cells. ^#^indicates a significant difference between control and curcumin-treated HepG2 cells without palmitate. α indicates a significant difference (*P* < 0.05) between PA without curcumin and PA-curcumin co-treated HepG2 cells.

To corroborate that oxidative stress is a causative agent of IL-8 up-regulation in palmitate-treated Sc and Gi HepG2 cells, anti-oxidative enzyme glutathione peroxidase (GPX) was examined for its effect on IL-8 level. As shown in Figure 7a, exogenous addition of GPX did not affect IL-8 secretion in Sc and Gi HepG2 cells compared with untreated control cells (*P* = 0.18, *P* = 0.25 respectively). Despite GPX did not affect palmitate-induced IL-8 in Sc HepG2 cells (*P* = 0.35), GPX partially suppressed palmitate-induced IL-8 (21% decrease, *P* < 0.05) in Gi HepG2 cells compared with that in palmitate-treated Gi HepG2 cells without GPX (Figure 7a). The finding that there is a significant difference between Sc and Gi cells in palmitate/GPX co-treatment but not in GPX treatment alone, indicating that G6PD is required for lowering pro-inflammatory cytokine secretion caused by excess ROS due to palmitate stimulation.

On the other hand, curcumin was used as an anti-inflammatory agent in this study. As shown in Figure 7b, curcumin treatments (50 and 100 μM) significantly reduced the IL-8 level of Sc and Gi HepG2 cells without palmitate treatment (50 μM: Sc, 71% decrease, Gi, 67% decrease; 100 μM: Sc, 70% decrease, Gi, 61% decrease, *P* < 0.05) compared with control cells without palmitate treatment (Figure 7b). Similarly, the IL-8 level of Sc and Gi HepG2 cells with palmitate treatment was significantly decreased by 50 μM curcumin (Sc, 55% decrease, Gi, 67% decrease, *P* < 0.05) when compared with that of palmitate-treated cells without curcumin. Further inhibition of palmitate-induced IL-8 in both cells occurred when treated with 100 μM curcumin (Sc, 84% decrease; Gi, 80% decrease, *P* < 0.05) compared to that of palmitate-treated cells without curcumin. These data demonstrate that curcumin significantly suppresses palmitate-induced IL-8 level in a dose dependent manner in both Sc and Gi HepG2 cells.

## Discussion

As the culprit causing many abnormal cellular functions, G6PD deficiency is well-known for inducing redox imbalance [2,3,6,7,10,11,31,35], which exacerbates cellular inflammatory response during disease progression [36,37]. Spolarics et al. have shown that ROS detoxifying enzymes, including G6PD, superoxide dismutase (SOD) and glutathione peroxidase (GPX) are up-regulated in hepatic endothelial cells of LPS-treated rats [38]. The increased antioxidant capacity indicates a protective role of G6PD against oxidative stress during hepatic inflammatory response [39]. Using G6PD-knockdown HepG2 cells, we have demonstrated in this study that elevated IL-8 secretion is paralleled with enhanced production of ROS (Figure 5). Such IL-8 elevation can be further enhanced by externally applied H_2_O_2_ (Figure 6). Our finding of ROS-induced IL-8 is in accord with previous reports in MKN28 cells [40] and HepG2 cells [41].

H_2_O_2_ is one of the well-known ROS species that modulates many signaling pathways [42]. H_2_O_2_ activates tumor necrosis factor-alpha (TNF-α) induced-NF-κB, whereas the antioxidant *N*-acetyl-*L*-cysteine (NAC) suppresses TNF-α induced-NF-κB [40]. G6PD can activate NF-κB in different cell types, including adipocytes in obesity and β-cells in type-two diabetes [33,34]. Indeed, NF-κB is involved in the ROS-induced IL-8 transcription [43]. Additional support to the notion that NF-κB is involved in the up-regulation of IL-8 either by palmitate treatment or G6PD knockdown in HepG2 cells comes from our study using the NF-κB inhibitor (BAY11-7085), which decreases IL-8 secretion at both basal and palmitate treated conditions (Figure 4a). The fact that knockdown of NF-κB subunit p65 by SiRNA causes a marked drop in IL-8 level (Figure 4c-d) further implicates a close relationship between NF-κB signaling and G6PD deficiency-derived oxidative stress in inflammation.

Antioxidants have been shown to exert the inhibitory effect on oxidative stress and inflammatory response caused by NF-κB activation [43-46]. In our current study, the exogenous addition of ROS scavenging enzyme GPX partially suppresses the up-regulation of IL-8 in palmitate-treated G6PD-knockdown HepG2 cells (Figure 7a). The suppression of IL-8 by GPX is similar to a previous finding that ROS scavenging agent NAC or dimethylsulfoxide (DMSO) inhibits IL-8 expression induced by TNF-α or IL-1β [40]. The possible explanation of IL-8 suppression by external addition of GPX in the culture medium is that GPX can protect against intracellular H_2_O_2_ readily crosses cell membrane similar to the action of catalase [47]. Thus extracellular GPX can dissipate H_2_O_2_ out of the cells and down-regulate H_2_O_2_-induced IL-8.

Curcumin (diferuloylmethane) is the pharmacologically active ingredient found in the spice turmeric (*Curcuma longa*) [48]. Recent studies suggest that curcumin exhibits anti-oxidative [49-51] and anti-inflammatory [52-55] activities. In addition, curcumin modulates several transcription factors, including AP-1, PPAR-γ, STAT, Nrf-2 and Wnt/β-catenin, [56], protein kinases MAP kinase p38 and ERK [57,58] and inflammatory cytokines TNF-α, IL-6 and IL-1β [59,60]. In our current study, we show that curcumin effectively reduces palmitate-induced IL-8 mRNA level (Figure 7b). Curcumin can ameliorate pro-inflammatory cytokine production [61], however, it has been reported that exposure of curcumin significantly induces apoptosis and cytochrome *c* release in HepG2 cells as early as 6 hours after treatment [62]. In our experimental condition, IL-8 secretion by short term palmitate-treated HepG2 cells is too low to be detected, whereas significantly increased IL-8 mRNA level in palmitate-treated HepG2 cells can be detected at 6 hours. Hence, we determined the effect of curcumin on IL-8 level in palmitate-treated HepG2 cells at 6 hours by qRT-PCR instead of ELISA.

The inactivation of NF-κB is a well-established mechanism of curcumin described in the literature [55,57,63,64]. It has been shown that curcumin suppresses the phosphorylation of IκBα (nuclear factor of kappa light polypeptide gene enhancer in B-cells inhibitor, alpha) through inactivation of IKK (IkappaB kinase) activity [65]. Moreover, curcumin down-regulates the expression of pro-inflammatory gene products regulated by NF-κB, including IL-8, through inhibiting IKK activity in intestinal epithelial cells [66]. A recent study in sepsis-induced acute lung injury rats shows that curcumin significantly enhances SOD activity and reduces lipid peroxidation in the lung [51]. Furthermore, curcumin down-regulates inflammatory cytokines TNF-α, IL-8 and MIF levels in the lung, suggesting a protective role in counteracting inflammation through down-regulation of pro-inflammatory cytokines and oxidative stress. Given that curcumin exerts its inhibitory actions through multiple targets, it is reasonable to speculate that curcumin may act as a non-specific anti-inflammatory agent in our study. Such speculation may justify its superior IL-8 inhibition capacity compared with GPX and NF-κB inhibitor in this study.

Several reports suggest that G6PD deficiency modulates cytokine response during inflammatory and immune responses. In G6PD mutant endotoxemic mice, altered cytokines, including elevated blood IL-6 level, has been documented [67,68]. Clinical studies have indicated that G6PD deficiency correlates with increased incidence of sepsis [69,70]. Moreover, diminished IL-10 and IFN-γ and increased IL-6 are present in African and Mediterranean forms of G6PD-deficient trauma patients [71]. Similarly, reduced monocyte IL-10 in G6PD-deficient trauma patients has been documented [72]. In contrast to the findings in G6PD-deficient adults, a more recent study has reported that the toll-like receptor (TLR) agonists-induced cytokine response in peripheral blood mononuclear cells (PBMCs) isolated from G6PD-deficient infants, including TNF-α, IL-6 and IL-10, is not different from PBMCs of G6PD normal subjects [73]. The discrepancy between G6PD-deficient adults and infants may lie in the relative immature innate immune response during infancy [74,75]. Additionally, the age of the subjects may also contribute to the disparity, because G6PD activity has been suggested to be inversely proportional to age [76].

## Conclusion

We have found that the secretion of pro-inflammatory cytokine IL-8 is most significantly increased in G6PD-deficient HepG2 cells by utilizing a cytokine array. Adopting a palmitate-induced inflammation HepG2 cell model, we have found that G6PD deficiency exacerbates pro-inflammatory cytokine IL-8 secretion in HepG2 cells. Mechanistically, G6PD deficiency up-regulates IL-8 through oxidative stress and NF-κB pathway. The palmitate-induced inflammation in G6PD-deficient HepG2 cells could serve as an in vitro model to study the role of altered redox homeostasis in chronic hepatic inflammation.

## Additional files

Additional file 1:
**Supplementary methods, reference and figure legends.**
Additional file 2: Figure S1. G6PD knockdown reduced G6PD activity and expression in HepG2 cells. (a) The G6PD activity of G6PD-scramble (Sc) and G6PD-knockdown (Gi) HepG2 cells were determined by enzymatic assay. The unit was expressed as U/mg of protein lysate. These results were representative of at least three separate experiments. *indicates a significant difference (P<0.05) between Sc and Gi HepG2 cells. (b) G6PD protein expression of Sc and Gi HepG2 cells were detected by Western blotting, the amount of G6PD protein was normalized to Actin in the respective sample. The blot shown was a representative of three separate experiments. Additional file 3: Figure S2. The morphology of palmitate-treated HepG2 cells. The morphology of control and palmitate-treated (0.3 mM) Sc and Gi HepG2 cells were visualized by Sudan Red (orange) and hematoxylin staining (blue). Additional file 4: Figure S3. The effect of palmitate treatment on lipid accumulation in HepG2 cells. The lipid levels of control and palmitate-treated (0.3 mM) Sc and Gi HepG2 cells were quantified by flow cytometry after Nile Red staining. These results were representative of at least three separate experiments. # indicates significant difference (P<0.05) between control and palmitate treatment. Additional file 5: Figure S4. Cytokine profile of Sc and Gi HepG2 cells with or without 0.3 mM of palmitate treatment for 24 hr. The result was a representative of two separate experiments. The normalization of relative cytokine level was described in the method section. The quantification result was shown in Table 2. Additional file 6: Table S1. Format of cytokine array.

## Abbreviations

G6PD: Glucose 6-phosphate dehydrogenase
ROS: Reactive oxygen species
IL-8: Interleukin-8
NADPH: Nicotinamide adenine dinucleotide phosphate
ELISA: Enzyme-linked immunosorbent assay
mRNA: Messenger RNA
qRT-PCR: Quantitative real time polymerase chain reaction
SiRNA: Small interference RNA
DCFDA: 2′, 7′ dichlorodihydrofluoroscein diacetate
H_2_O_2_: Hydrogen peroxide
GPX: Glutathione peroxidase
NF-κB: Nuclear factor-kappa B
