# Supplementary material for: Glucose 6-phosphate dehydrogenase knockdown enhances IL-8 expression in HepG2 cells via oxidative stress and NF-κB signaling pathway
Source: J Inflamm (Lond). 2015 Apr 24;12:34. doi: 10.1186/s12950-015-0078-z (PMC4419400; doi:10.1186/s12950-015-0078-z)
Supplement: Additional file 1: — Supplementary methods, reference and figure legends. [file 12950_2015_78_MOESM1_ESM.pdf]

Supplementary Materials

1. Methods

1.1 G6PD activity assay

The G6PD activity in HepG2 cells was determined spectrophotometrically at 340 nm by the reduction of $NADP^+$ in the presence of glucose 6-phosphate, which has been described previously (Lin, et al. 2010).

1.2 Microscopic visualization of lipid accumulation by Sudan Red staining

The visualization of lipid accumulation in palmitate-treated Sc and Gi HepG2 cells stained by lipid dye Sudan Red and nuclear dye Hematoxylin was modified from a previous protocol (Jeney, et al. 2000). In brief, palmitate-treated HepG2 cells were fixed by 3.7% formaldehyde followed by rinse with 60% isopropanol. The cellular lipid was stained with 0.3% Sudan Red solution (Alfa Aesar, MA, USA) for 15 min and the nuclei was counterstained with Hematoxylin (Sigma). The microscopic images of cells were taken by using Zeiss LSM 510 Meta System (Carl Zeiss MicroImaging, GmbH, Heidelberg, Germany)

1.3 Lipid measurement by flow cytometry

The quantification of lipid in palmitate-treated Sc and Gi HepG2 cells was analyzed by flow cytometry modified from a previous protocol (Gomez-Lechon, et al. 2007). In brief, palmitate-treated cells were incubated with 0.25 mg/ml Nile Red solution for 30 min at 37°C followed by treatment of Trypsin-EDTA (Invitrogen, CA, USA). The trypsinized cells were analyzed for lipid content by a FACS Calibur flow cytometer (excitation 488 nm, emission 550 nm) (Becton Dickson Biosciences, CA, USA). The flow cytometry data was analyzed by Cell Quest Pro software (Becton Dickson Biosciences).

2. Supplementary reference

Gomez-Lechon MJ, Donato MT, Martinez-Romero A, Jimenez N, Castell JV, O'Connor JE. A human hepatocellular in vitro model to investigate steatosis. Chem Biol Interact 165(2), 106-16, 2007.

Jeney F, Bazso-Dombi E, Oravecz K, Szabo J, Nagy IZ. Cytochemical studies on the fibroblast-preadipocyte relationships in cultured fibroblast cell lines. Acta Histochem 102(4), 381-9, 2000.

Lin CJ, Ho HY, Cheng ML, You TH, Yu JS, Chiu DT. Impaired dephosphorylation renders G6PD-knockdown HepG2 cells more susceptible to H(2)O(2)-induced apoptosis. Free Radic Biol Med 49(3), 361-73, 2010.

3. Supplementary figure captions

Figure S1. G6PD knockdown reduced G6PD activity and expression in HepG2 cells. (a) The G6PD activity of G6PD-scramble (Sc) and G6PD-knockdown (Gi) HepG2 cells were determined by enzymatic assay. The unit was expressed as U/mg of protein lysate. These results were representative of at least three separate experiments. * indicates a significant difference ($P<0.05$) between Sc and Gi HepG2 cells. (b) G6PD protein expression of Sc and Gi HepG2 cells were detected by Western blotting, the amount of G6PD protein was normalized to Actin in the respective sample. The blot shown was a representative of three separate experiments.

Figure S2. The morphology of palmitate-treated HepG2 cells. The morphology of control and palmitate-treated (0.3 mM) G6PD-scramble (Sc) and G6PD-knockdown (Gi) HepG2 cells were visualized by Sudan Red (orange) and hematoxylin staining (blue).

Figure S3. The effect of palmitate treatment on lipid accumulation in HepG2 cells. The lipid levels of control and palmitate-treated (0.3 mM) Sc and Gi HepG2 cells were quantified by flow cytometry after Nile Red staining. These results were

representative of at least three separate experiments. # indicates significant difference (P<0.05) between control and palmitate treatment.

Figure S4. Cytokine profile of Sc and Gi HepG2 cells with or without 0.3 mM of palmitate treatment for 24 hr. The result was a representative of two separate experiments. The normalization of relative cytokine level was described in the method section. The quantification result was shown in Table 2.
