# Supplementary figures and images for: Glucose 6-phosphate dehydrogenase knockdown enhances IL-8 expression in HepG2 cells via oxidative stress and NF-κB signaling pathway
Source: J Inflamm (Lond). 2015 Apr 24;12:34. doi: 10.1186/s12950-015-0078-z (PMC4419400; doi:10.1186/s12950-015-0078-z)

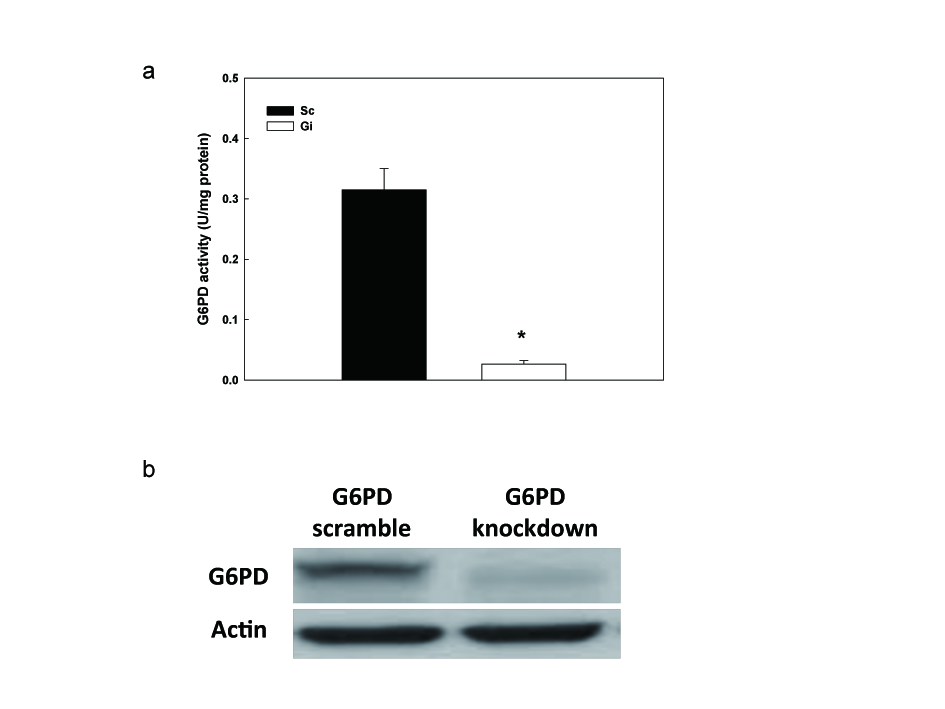

Supplement: Additional file 2: Figure S1. — G6PD knockdown reduced G6PD activity and expression in HepG2 cells. (a) The G6PD activity of G6PD-scramble (Sc) and G6PD-knockdown (Gi) HepG2 cells were determined by enzymatic assay. The unit was expressed as U/mg of protein lysate. These results were representative of at least three separate experiments. *indicates a significant difference (P<0.05) between Sc and Gi HepG2 cells. (b) G6PD protein expression of Sc and Gi HepG2 cells were detected by Western blotting, the amount of G6PD protein was normalized to Actin in the respective sample. The blot shown was a representative of three separate experiments. [file 12950_2015_78_MOESM2_ESM.tiff]

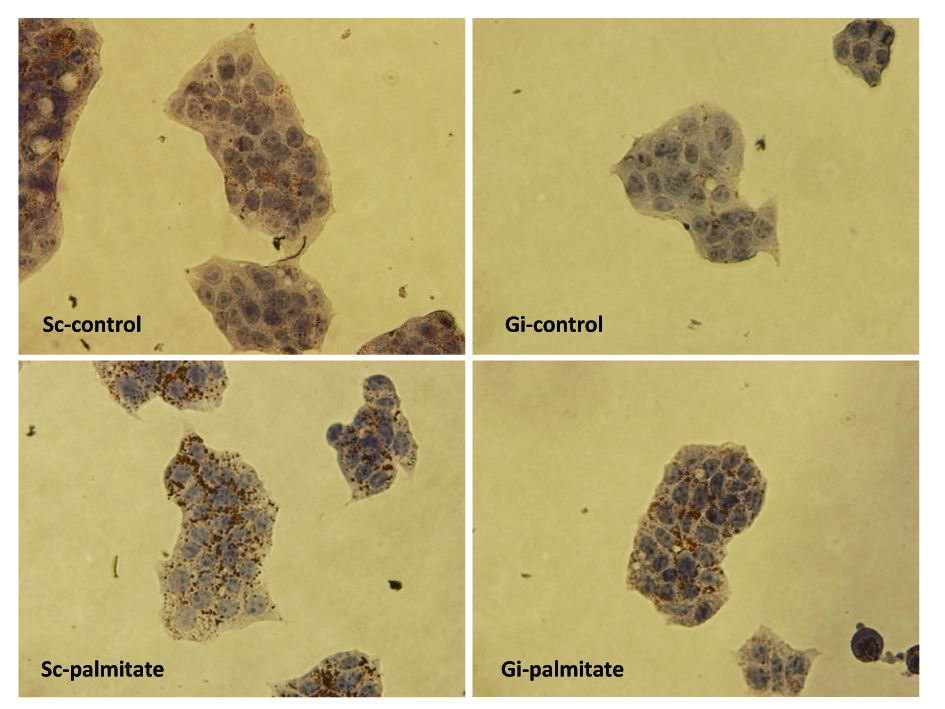

Supplement: Additional file 3: Figure S2. — The morphology of palmitate-treated HepG2 cells. The morphology of control and palmitate-treated (0.3 mM) Sc and Gi HepG2 cells were visualized by Sudan Red (orange) and hematoxylin staining (blue). [file 12950_2015_78_MOESM3_ESM.tiff]

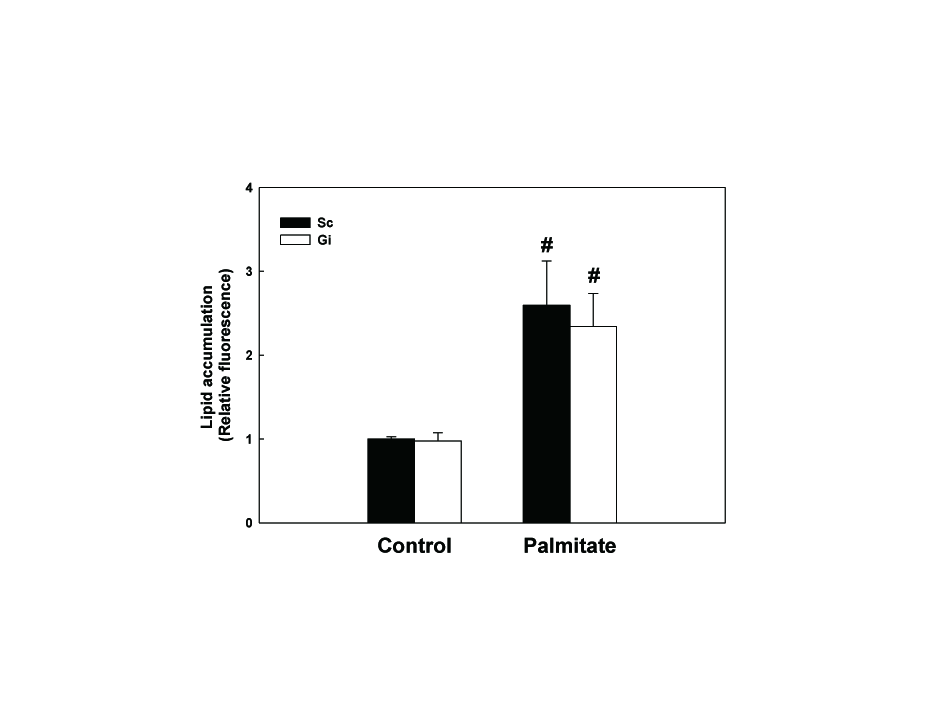

Supplement: Additional file 4: Figure S3. — The effect of palmitate treatment on lipid accumulation in HepG2 cells. The lipid levels of control and palmitate-treated (0.3 mM) Sc and Gi HepG2 cells were quantified by flow cytometry after Nile Red staining. These results were representative of at least three separate experiments. # indicates significant difference (P<0.05) between control and palmitate treatment. [file 12950_2015_78_MOESM4_ESM.tiff]

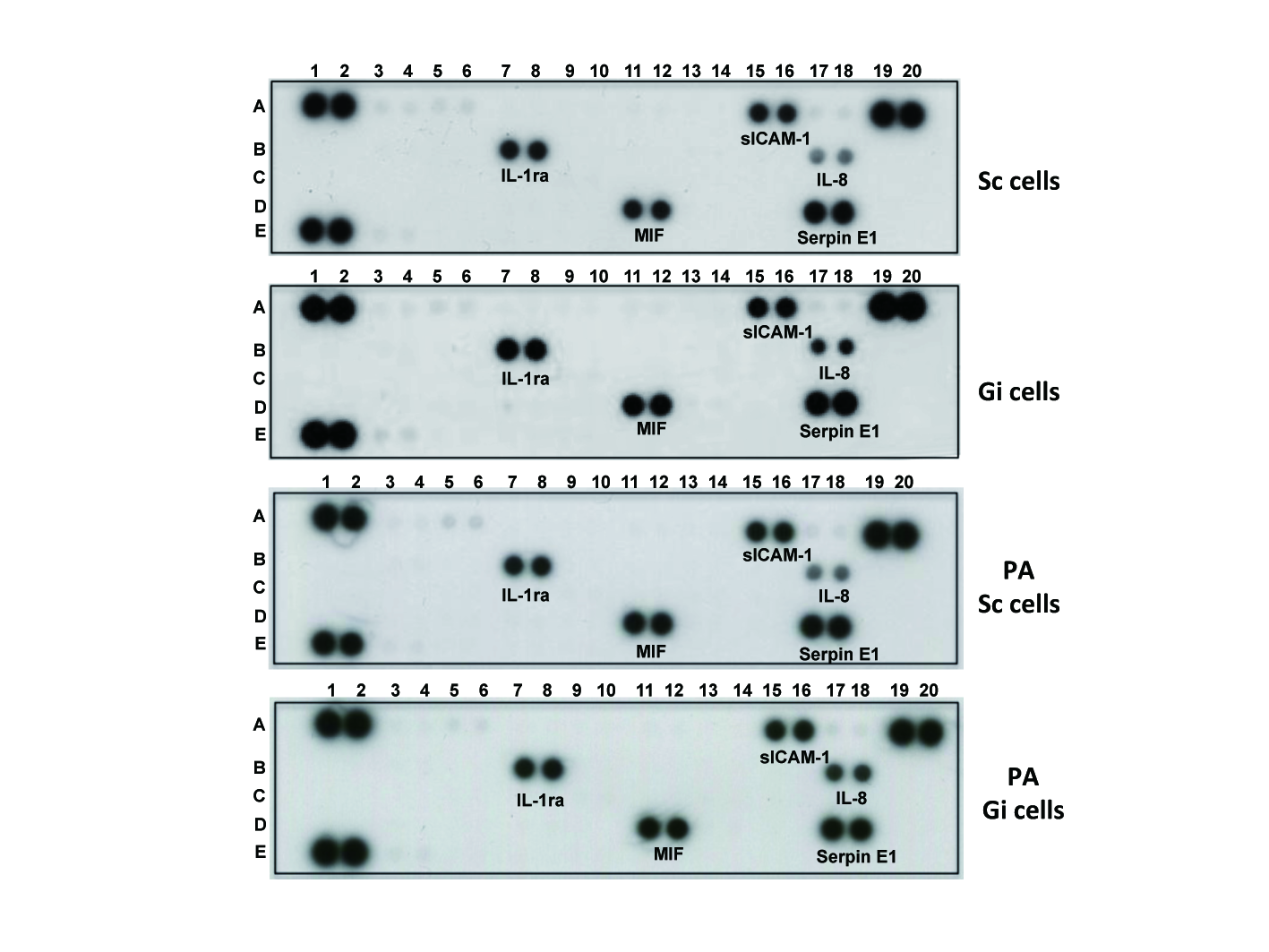

Supplement: Additional file 5: Figure S4. — Cytokine profile of Sc and Gi HepG2 cells with or without 0.3 mM of palmitate treatment for 24 hr. The result was a representative of two separate experiments. The normalization of relative cytokine level was described in the method section. The quantification result was shown in Table 2. [file 12950_2015_78_MOESM5_ESM.tiff]
