# Supplementary material for: Glucose 6-phosphate dehydrogenase knockdown enhances IL-8 expression in HepG2 cells via oxidative stress and NF-κB signaling pathway
Source: J Inflamm (Lond). 2015 Apr 24;12:34. doi: 10.1186/s12950-015-0078-z (PMC4419400; doi:10.1186/s12950-015-0078-z)
Supplement: Additional file 6: Table S1. — Format of cytokine array. [file 12950_2015_78_MOESM6_ESM.pdf]

Table S1 Cytokine array format

Coordinate	Target/Control
A1, A2	Positive Control
A3, A4	C5a
A5, A6	CD40 Ligand
A7, A8	G-CSF
A9, A10	GM-CSF
A11, A12	GROα
A13, A14	I-309
A15, A16	sICAM-1
A17, A18	IFN-γ
A19, A20	Positive Control
B3, B4	IL-1α
B5, B6	IL-1β
B7, B8	IL-1ra
B9, B10	IL-2
B11, B12	IL-4
B13, B14	IL-5
B15, B16	IL-6
B17, B18	IL-8
C3, C4	IL-10
C5, C6	IL-12 p70
C7, C8	IL-13
C9, C10	IL-16
C11, C12	IL-17
C13, C14	IL-17E
C15, C16	IL-23
C17, C18	IL-27
D3, D4	IL-32α
D5, D6	IP-10
D7, D8	I-TAC
D9, D10	MCP-1
D11, D12	MIF
D13, D14	MIP-1α
D15, D16	MIP-1β
D17, D18	Serpin E1
E1, E2	Positive Control
E3, E4	RANTES
E5, E6	SDF-1
E7, E8	TNF-α
E9, E10	sTREM-1
E19, E20	Negative Control
